
JOURNAL INFORMATION
==============================
NLM Title Abbreviation: ScientificWorldJournal
Journal ID: ScientificWorldJournal
NLM Journal ID: 101131163
Journal ID: TSWJ

The Scientific World Journal

ISSN: 2356-6140
EISSN: 1537-744X
Publisher: Wiley

ARTICLE INFORMATION
==============================
PMCID: PMC5917322
PMID: 17041718
DOI: 10.1100/tsw.2006.230
Article ID: 483938
Article version: 1
Subjects: Directions in Science

xSyndecan-4 Regulates Gastrulation and Neural Tube Closure in Xenopus Embryos

Muñoz Rosana
Larraín Juan *
Department of Cell and Molecular Biology, Center for Cell Regulation and Pathology, Millennium Nucleus in Developmental Biology, Faculty of Biological Sciences, P. Universidad Católica de Chile, Alameda 340, Santiago, Chile
*Juan Larraín: jlarrain@bio.puc.clAcademic Editor: Martin Goette

Publication date: 2006
Electronic publication date: 2006 Oct 9
Volume: 6
First page: 1298
Last page: 1301
Received 2006 Aug 21; Revised 2006 Sep 22; Accepted 2006 Sep 28
Copyright: Copyright © 2006 Rosana Muñoz and Juan Larraín.
Copyright year: 2006
License: This is an open access article distributed under the Creative Commons Attribution License, which permits unrestricted use, distribution, and reproduction in any medium, provided the original work is properly cited.
License URL: https://creativecommons.org/licenses/by/3.0/

Keywords: Syndecan, convergent and extension movements, neural tube closure, neural tube defects, heparan sulfate proteoglycan

Funding: Fondo Nacional de Ciencia y Tecnología 1030481 Center for Cell Regulation and Pathology Millennium Nucleus in Developmental Biology

==============================
Directions in Science
*TheScientificWorldJOURNAL* (2006) 6, 1298-1301
ISSN 1537-744X; DOI 10.1100/tsw.2006.230

TheScientificWorldJOURNAL
www.thescientificworld.com

### xSyndecan-4 Regulates Gastrulation and Neural Tube Closure in *Xenopus* Embryos

Rosana Muñoz and Juan Larraín*

*Department of Cell and Molecular Biology, Center for Cell Regulation and Pathology, Millennium Nucleus in Developmental Biology, Faculty of Biological Sciences, P. Universidad Católica de Chile, Alameda 340, Santiago, Chile*

**E-mail**: jlarrain@bio.puc.cl

*Received August 21, 2006; Revised September 22, 2006; Accepted September 28, 2006; Published October 9, 2006*

---

**KEYWORDS**: Syndecan, convergent and extension movements, neural tube closure, neural tube defects, heparan sulfate proteoglycan, *Xenopus laevis*

---

Gastrulation is the morphogenetic process in embryos of multicellular organisms by which the presumptive mesoderm and endoderm move inside the ectoderm to form a three-layered embryo[1]. Once gastrulation is completed, a process known as neurulation is responsible for correct formation of the neural tube, the embryonic precursor of the brain and spinal cord. The proper neural plate shape is acquired during gastrulation, in part, through a process named convergent extension (CE). CE involves active cell movements and rearrangements that are driven by intercalation of polarized cells and regulated, among others, by noncanonical Wnt signaling[2,3,4]. Failures during these movements result in severe congenital malformations, collectively known as neural tube defects (NTD). Spinal NTD include myelomeningocele (bifid spine), the severity of which varies, but can cause prenatal lethality and severe neurological deficit in surviving individuals. In humans, NTD are relatively common with a frequency of 0.2–3.5 per 1,000 pregnancies[5].

Genetic studies in *Drosophila*, mice, and zebrafish have begun to reveal specific functions for heparan sulfate proteoglycans (HSPGs) throughout embryonic development[6]. Although there is no clear evidence about a possible role of HSPGs during neural tube closure, reports on zebrafish and *Xenopus* embryos have suggested that HSPGs are involved in gastrulation movements[7,8]. In our laboratory, we have started to study the function of proteoglycans (PGs) in early patterning of the vertebrate embryo. We have demonstrated that biglycan, a chondroitin sulfate PG, regulates dorsal-ventral patterning through modulation of BMP4 signaling activity[9]. Our recent paper published in *Nature Cell Biology* (Muñoz, R., Moreno, M., Oliva, C., Orbenes, C., and Larraín, J. **8(5),** 492–500. 2006) shows that a cell-surface HSPG, Syndecan-4 (Syn4), is critical during *X. laevis* gastrulation and neural tube closure. At the gastrula stage, xSyn4 is expressed in the involuting dorsal mesoderm and the anterior neuroectoderm, both tissues that undergo CE. Both gain- and loss-of-function of xSyn4 impaired CE in *Xenopus* embryos, resulting in failure of neural tube closure (Fig. 1). Several studies have demonstrated that a functional noncanonical Wnt signaling is required for proper CE. This pathway regulates the polarized protrusive activity of dorsal mesodermal cells and either its activation or inactivation results in disruption of proper cell polarization and CE[10,11,12,13,14,15]. In accordance with a possible role of xSyn4 in noncanonical Wnt signaling, this paper demonstrates that xSyn4 is able to interact biochemically and functionally with the Wnt receptor

*Corresponding author.
©2006 with author.
Published by TheScientificWorld, Ltd.; www.thescientificworld.com

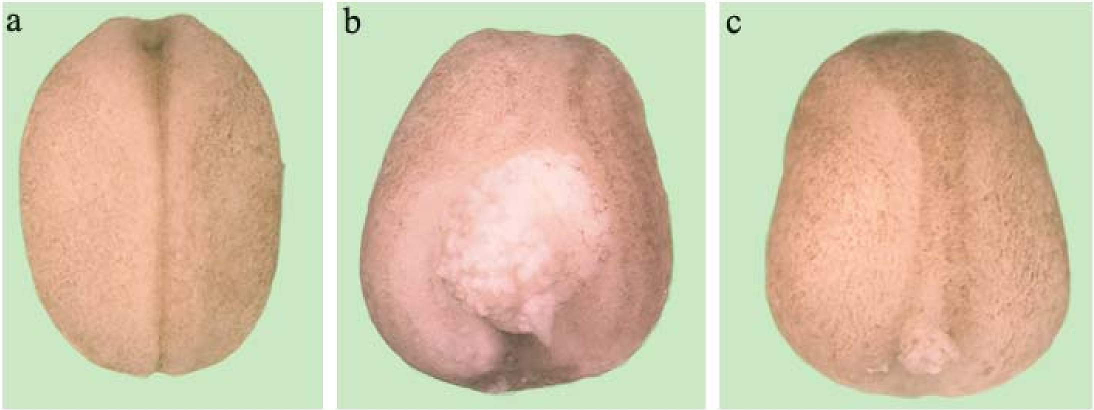

**FIGURE 1.** xSyn4 is essential for CE. Microinjection of morpholinos for xSyn4 (xSyn4MO) caused severe defects in gastrulation and neural tube closure in contrast with control embryos (a). The phenotypes induced by xSyn4MO were classified into those with severe gastrulation defects (b) and those with only neural tube closure defects (c).

Frizzled7 (xFz7) and its downstream effector Dishevelled (xDsh). Furthermore, xSyn4 is necessary and sufficient for translocation of xDsh to the plasma membrane, a hallmark of Wnt signaling activation. However, it has been suggested that the ability of xSyn4 to translocate xDsh is regulated by fibronectin. Syn4 binds to the heparin-binding domain of fibronectin through its glycosaminoglycan chains (GAG)[16] and is required, together with integrins, for proper stabilization of focal adhesion sites[17,18,19]. Recent reports have demonstrated that chronic depletion of fibronectin and acute disruptions of $\alpha 5\beta 1$ integrin-fibronectin binding increases the frequency and randomizes the orientation of polarized cellular protrusions. Consequently, it has been suggested that integrin-fibronectin interactions normally repress frequent random protrusions in favor of fewer mediolaterally oriented ones. In the absence of $\alpha 5\beta 1$ integrin binding to fibronectin, CE still occurs, but results in convergent thickening instead of extension[20].

In conclusion, we have demonstrated by gain- and loss-of-function assays that xSyn4 regulates and is essential for proper CE during early development of *Xenopus* embryos. We also showed that xSyn4 is a component of the noncanonical Wnt pathway, being necessary for translocation of xDsh to the plasma membrane, a process that requires its cytoplasmic domain. However, this function may be done by at least two mechanisms: xSyn4 interacts directly with xDsh or through complex with xFz7 (Fig. 2). Importantly, the ability of xSyn4 to interact with xDsh is regulated by fibronectin. Together, these results suggest a model where specific activation of the noncanonical Wnt pathway results from the activation of xSyn4 by fibronectin and xFz7 by Wnt ligands.

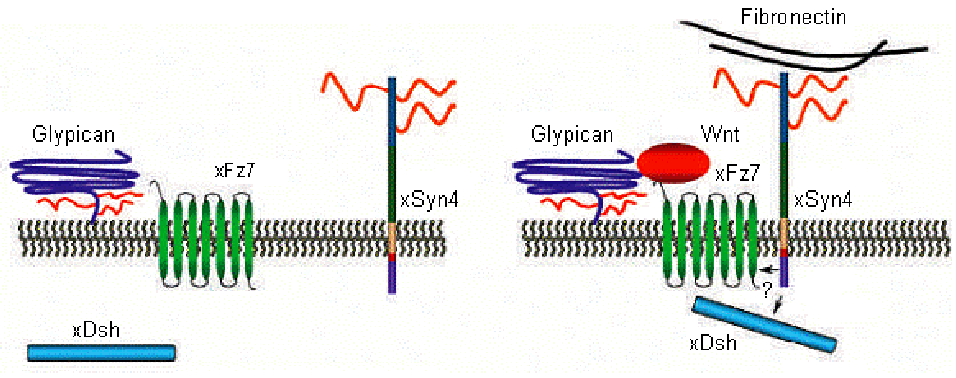

**FIGURE 2.** Schematic representation of the proposed role of xSyn4 in noncanonical Wnt signaling.

Two models have been proposed to explain how HSPGs modulate Wingless/Wnt signaling. In the first model, HSPGs are necessary for maintaining the local concentration of Wingless/Wnt ligand that is available for its receptor[21,22]. In the second model, HSPGs are proposed to act as coreceptors that directly facilitate the formation of Wingless/Wnt -Fz signaling complexes[23,24]. Recently, a coreceptor function for glypican4 (xgly4), a cell-surface HSPG, in CE movement and noncanonical Wnt signaling, has also been described in *Xenopus* embryos[7]. Xgly4 interacts physically with Wnt11, and this interaction results critical for activation of Wnt/PCP pathway[8]. Although it is not yet clear how GAGs and core protein of HSPG affects signaling, our experiments suggest that GAGs are important for interaction with fibronectin and the protein core for complexing with xFz7 and translocation of xDsh to plasma membrane (data not shown). It is also interesting to mention that a role for HSPGs in the modulation of canonical Wnt signaling during axis determination of early *Xenopus* embryos has been reported[25]. These, together with our findings, suggest a role for HSPG in the specific activations of the different branches of Wnt signaling.

Recently, the requirement for CE during the initiation of neural tube closure has become clear. Mouse mutants named *loop-tail*, *crash* and *circletail*, and *dishevelled-1*, *dishevelled-2* double knockdown, showed improper neural tube closure, which leads to craniorachischisis (type of NTDs). Each of the mutant genes in which craniorachischisis is observed encodes a protein that functions in a branch of noncanonical Wnt signaling: the so-called planar cell polarity pathway. The cause of craniorachischisis in these mutants has been inferred to be a failure of CE, because, in *Xenopus*, overexpression of wild-type or mutant forms of xDsh and Strabismus (which blocks convergent extension) usually results in similar neural tube closure defects[5,25]. It is important to note that overexpression and morpholino knockdown of xSyn4 produces open NTD in *Xenopus* embryos. We propose that Syn4 works as a coreceptor for specific activation of the noncanonical Wnt pathway.

### ACKNOWLEDGMENTS

We thank G. Reig for comments on the manuscript. This work was supported by Fondo Nacional de Ciencia y Tecnología (FONDECYT, 1030481). Center for Cell Regulation and Pathology (FONDAP). Millennium Nucleus in Developmental Biology (MNDB).

### REFERENCES

1. Keller, R. (2005) Cell migration during gastrulation. *Curr. Opin. Cell Biol.* **17**, 533–541.
2. Keller, R. (2002) Shaping the vertebrate body plan by polarized cell movements. *Science* **298**, 1950–1954.
3. Solnica-Krezel, L. (2005) Conserved patterns of cell movements during vertebrate gastrulation. *Curr. Biol*. **15**, R213–R228.
4. Wallingford, J.B., Fraser S.E., and Harland, R.M. (2002) Convergent extension: the molecular control of polarized cell movement during embryonic development. *Dev. Cell* **2,** 695–706.
5. Greene, N. and Copp, A. (2005) Mouse models of neural tube defects: investigating preventive mechanisms. *Am. J. Med. Genet. C (Semin. Med. Genet.)* **135C**, 31–41.
6. Perrimon, N. and Bernfield, M. (2000) Specificities of heparan sulphate proteoglycans in developmental processes. *Nature* **404**, 725–728.
7. Topczewski, J., Sepich, D.S., Myers, D.C., Walker, C., Amores, A., Lele, Z., Hammerschmidt, M., Postlethwait, J., and Solnica-Krezel, L. (2001) The zebrafish glypican knypek controls cell polarity during gastrulation movements of convergent extension. *Dev. Cell* **1**, 251–264.
8. Ohkawara, B., Yamamoto, T., Tada, M., and Ueno, N. (2003) Role of glypican 4 in the regulation of convergent extension movements during gastrulation in Xenopus laevis. *Development* **130**, 2129–2138.
9. Moreno, M., Muñoz, R., Aroca, F., Labarca, M., Brandan, E., and Larraín, J. (2005) Biglycan is a new extracellular component of the Chordin-BMP4 signalling pathway. *EMBO J*. **24**, 1397–1405.
10. Djiane, A., Riou, J., Umbhauer, M., Boucaut, J., and Shi, D. (2000) Role of frizzled-7 in the regulation of convergent extension movements during gastrulation in *Xenopus laevis*. *Development* **127**, 3091–3100.
11. Tada, M. and Smith, J.C. (2000) Xwnt11 is a target of Xenopus brachyury: regulation of gastrulation movements via dishevelled but not through the canonical Wnt pathway. *Development* **127**, 2227–2238.

12. Wallingford, J.B. and Harland, R.M. (2001) Xenopus dishevelled signalling regulates both neural and mesodermal convergent extension: parallel forces elongating the body axis. *Development* **128**, 2581–2592.
13. Park, M. and Moon, R.T. (2002) The planar cell-polarity gene stbm regulates cell behaviour and cell fate in vertebrate embryos. *Nat. Cell Biol.* **4**, 20–25.
14. Goto, T. and Keller, R. (2002) The planar cell polarity gene strabismus regulates convergent and extension and neural fold closure in Xenopus. *Dev. Biol.* **247**, 165–181.
15. Wallingford, J.B. et al. (2000) Dishevelled controls cell polarity during Xenopus gastrulation. *Nature* **405**, 81–85.
16. Woods, A., Longley, R.L., Tumova, S., and Couchman, J.R. (2000) Syndecan-4 binding to the high affinity heparin-binding domain of fibronectin drives focal adhesion formation in fibroblasts. *Arch. Biochem. Biophys.* **374**, 66–72.
17. Woods, A. and Couchman, J.R. (2001) Syndecan-4 and focal adhesion function. *Curr. Opin. Cell Biol.* **13**, 578–583.
18. Marsden, M. and DeSimone, D. (2001) Regulation of cell polarity, radial intercalation and epiboly in *Xenopus*: novel roles for integrin and fibronectin. *Development* **128**, 3635–3647.
19. Marsden, M. and DeSimone, D. (2003) Integrin–ECM interactions regulate cadherin-dependent cell adhesion and are required for convergent extension in *Xenopus*. *Curr. Biol.* **13**, 1182–1191.
20. Davidson, L., Marsden, M., Keller, R., and DeSimone, W. (2006) Integrin a5b1 and fibronectin regulate polarized cell protrusions required for Xenopus convergence and extension. *Curr. Biol.* **16**, 833–844.
21. Hacker, U., Lin, X., and Perrimon, N. (1997) The Drosophila sugarless gene modulates Wingless signaling and encodes an enzyme involved in polysaccharide biosynthesis. *Development* **124**, 3565–3573.
22. Pfeiffer, S., Ricardo, S., Manneville, J.B., Alexandre, C., and Vincent, J.P. (2002) Producing cells retain and recycle Wingless in Drosophila embryos. *Curr. Biol.* **12**, 957–962.
23. Lin, X. and Perrimon, N. (1999) Dally cooperates with Drosophila Frizzled 2 to transduce Wingless signalling. *Natur*[illegible] 281–284.
24. Lin, X. (2004) Functions of heparan sulfate proteoglycans in cell signaling during development. *Development* **131**, [illegible] 6021.
25. Tao, Q., Yokota, C., Puck, H., Kofron, M., Birsoy, B., Yan, D., Asashima, M., Wylie, C.C., Lin, X., and Heasman, J. ([illegible] Maternal wnt11 activates the canonical Wnt signaling pathway required for axis formation in Xenopus embryos. *Cel*[illegible] 857–871.
26. Coop, A., Greene, N., and Murdoch, J. (2003) The genetic basis of mammalian neurulation. *Nat. Rev. Genet.* **4**, 784–79[illegible]

**This article should be cited as follows:**

Muñoz, R. and Larraín, J. (2006) xSyndecan-4 regulates gastrulation and neural tube closure in *Xenopus* embryos. *TheScientificWorldJOURNAL* **6**, 1298–1301. DOI 10.1100/tsw.2006.230.
